# Effect of crown to implant ratio and implantoplasty on the fracture resistance of narrow dental implants with marginal bone loss: an in vitro study

**DOI:** 10.1186/s12903-020-01323-z

**Published:** 2020-11-19

**Authors:** Bruno Leitão-Almeida, Octavi Camps-Font, André Correia, Javier Mir-Mari, Rui Figueiredo, Eduard Valmaseda-Castellón

**Affiliations:** 1 Faculty of Dental Medicine, Center for Inter-Disciplinary Research in Health (CIIS), Universidade Católica Portuguesa, Estrada da Circunvalação, 3504-505 Viseu, Portugal; 2 Oral Surgery and Implantology, Faculty of Medicine and Health Sciences, University of Barcelona, Barcelona, Spain

**Keywords:** Peri-implantitis, Dental implants, Compressive strength, Titanium, Implantoplasty

## Abstract

**Background:**

Peri-implantitis is a biological complication that affects soft and hard tissues around dental implants. Implantoplasty (IP) polishes the exposed implant surface, to decontaminate it and make it less prone to bacterial colonization. This study investigates whether a higher clinical crown-to-implant-ratio (CIR) reduces implant fracture resistance and whether implants are more fracture-prone after IP in the presence of 50% of bone loss.

**Methods:**

Forty-eight narrow platform (3.5 mm) 15 mm long titanium dental implants with a rough surface and hexagonal external connection were placed in standardized bone-like resin casts leaving 7.5 mm exposed. Half were selected for IP. The IP and control groups were each divided into 3 subgroups with different clinical CIRs (2:1, 2.5:1 and 3:1). The implant wall width measurements were calculated using the software ImageJ v.1.51 through the analysis of plain x-ray examination of all the samples using standardized mounts. A fracture test was performed and scanning electron microscopy was used to evaluate maximum compression force (F_max_) and implant fractures.

**Results:**

IP significantly reduced the implant wall width (*P* < 0.001) in all reference points of each subgroup. F_max_ was significantly higher in the 2:1 subgroup (control = 1276.16 N ± 169.75; IP = 1211.70 N ± 281.64) compared with the 2.5:1 (control = 815.22 N ± 185.58, *P* < 0.001; IP = 621.68 N ± 186.28, *P* < 0.001) and the 3:1 subgroup (control = 606.55 N ± 111.48, *P* < 0.001; IP = 465.95 N ± 68.57, *P* < 0.001). Only the 2.5:1 subgroup showed a significant reduction (*P* = 0.037) of the F_max_ between the controls and the IP implants. Most fractures were located in the platform area. Only 5 implants with IP of the 2:1 CIR subgroup had a different fracture location (4 fractures in the implant body and 1 in the prosthetic screw).

**Conclusions:**

IP significantly reduces the fracture resistance of implants with a 2.5:1 CIR. The results also suggest that the CIR seems to be a more relevant variable when considering the resistance to fracture of implants, since significant reductions were observed when unfavorable CIR subgroups (2.5:1 and 3:1 CIR) were compared with the 2:1 CIR samples.

## Background

Implant failure appears to have several causes: biological, mechanical or iatrogenic [1–3]. Peri-implantitis (PI) is one of the major concerns among clinicians, as it may affect 34% of patients and 21% of implants and lead to implant loss [4].

Several approaches to implant surface decontamination have been studied. They include air-powder abrasion, ultrasonic and manual debridement (using plastic, carbon stainless steel, graphite or titanium curettes), implantoplasty (IP), laser therapy and sterile saline rinses, among others [5–8]. Mechanical debridement has also been complemented by the use of a number of substances, such as citric acid, hydrogen peroxide, cetylpyridinium chloride, tetracycline, ethylenediamine tetraacetic acid or chlorhexidine [9]. IP is a common procedure that consists of polishing rough implant surfaces outside the bony envelope, making them less prone to bacterial accumulation, as surface roughness may be risk factor for peri-implant disease. IP is effective in the long term for arresting bone loss caused by PI, both alone and in combination with surgical regenerative procedures and does not seem to be associated with any biological or mechanical complication of importance [9–13]. However, thermal increases during the procedure that could affect the bone, lower resistance to fractures due to reducing the thickness of the implant walls, and the local and systemic biological repercussions that the dispersion of titanium particles might have in the long term have been signaled as potential problems of IP performance [14–19].

Increasing bone loss due to PI was shown to increase clinical crown-to-implant ratio (CIR), which, in turn, was reported to reduce the resistance to fracture of intact dental implants [20, 21]. Also, IP, which is often used as a part of the treatment of PI, reduces the thickness of the implant walls and might weaken the strength of implants [15]. Since the effect of the CIR on implants treated with IP has not been addressed yet, it would be of great interest to assess whether IP is a safe technique when implants with high CIRs are involved. Furthermore, since the maximum failure strength of bone level implants is expected to remain high after IP, narrow implants were selected to simulate an unfavorable scenario. Indeed, according to a recent report by Bertl et al., narrow diameter implants have a significant lower resistance strength compared with regular diameter implants [22].

The main study hypothesis was that a high CIR negatively affects the fracture resistance of narrow implants treated with IP in a situation of 50% bone loss. Therefore, the main objectives of this research were: (1) to analyze whether an increased CIR reduces the fracture resistance of implants with IP versus control implants, and (2) to assess whether implants subjected to IP are more prone to fracture in comparison with control implants, regardless of the CIR, in the presence of 50% bone loss. A secondary aim was to describe the changes in implant wall width after IP.

## Materials and methods

**Fig. 1:**
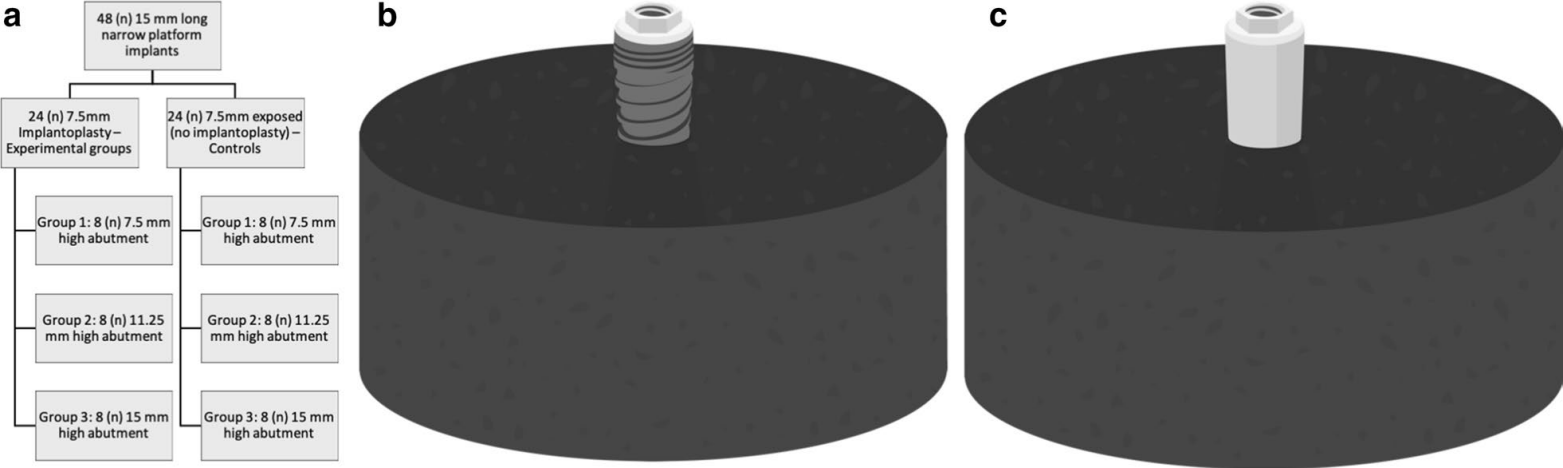
**a** Study design, groups and subgroups; **b** sample before IP; **c** sample after IP

An in vitro study was conducted using 48 type V titanium narrow platform implants, 3.5 mm in diameter and 15 mm long, with a rough surface and a hexagonal external connection (Ocean E.C., Avinent Implant System S.L., Santpedor, Spain). Half of the sample was randomly allocated to the IP group. The apical half of each implant was inserted, leaving 7.5 mm exposed, in standardized bone-like resin casts (EA 3471 A and B Loctite®, Henkel AG and Company, Düsseldorf, Germany) with a ≥ 3 GPa modulus of elasticity in accordance with International Organization for Standardization (ISO) standard 14801:2016 (third edition) [23]. Both groups were divided into 3 subgroups of 8 implants each, which received screwed hemispherical loading abutments of one of three heights: 7.5 mm, 11.25 mm and 15 mm, simulating clinical CIR of 2:1, 2.5:1 and 3:1, respectively (Fig. 1).

### Implantoplasty

IP of the exposed implant surface was performed using a high-speed air-powered hand piece (Bora Blackline LED, Bien-Air Dental SA, Langgasse, Switzerland) with an abutment protecting the connection. After removing the threads of the exposed portion of the implants, using an oval-shape tungsten carbide bur (H379 314,023; Komet Dental, Lemgo, Germany), the surface was polished with two-step silicon carbide polishers (9618,314,030 and 9608,314,030; Komet Dental, Lemgo, Germany) until it was macroscopically flat and smooth. A new set of burs was used for each sample. The procedure was performed by an experienced surgeon with 2.8 × magnification loupes (Galilean HD and Focus™ LED 6000 k, ExamVision ApS, Samsø, Denmark), under copious water irrigation and adequate light conditions, similar to a clinical scenario, although the cast was held by the operator and turned by hand. The time spent on each procedure was recorded. When the IP procedure was finished, the surface was cleaned with water and dried with air.

### Radiographic implant wall width measurements

**Fig. 2:**
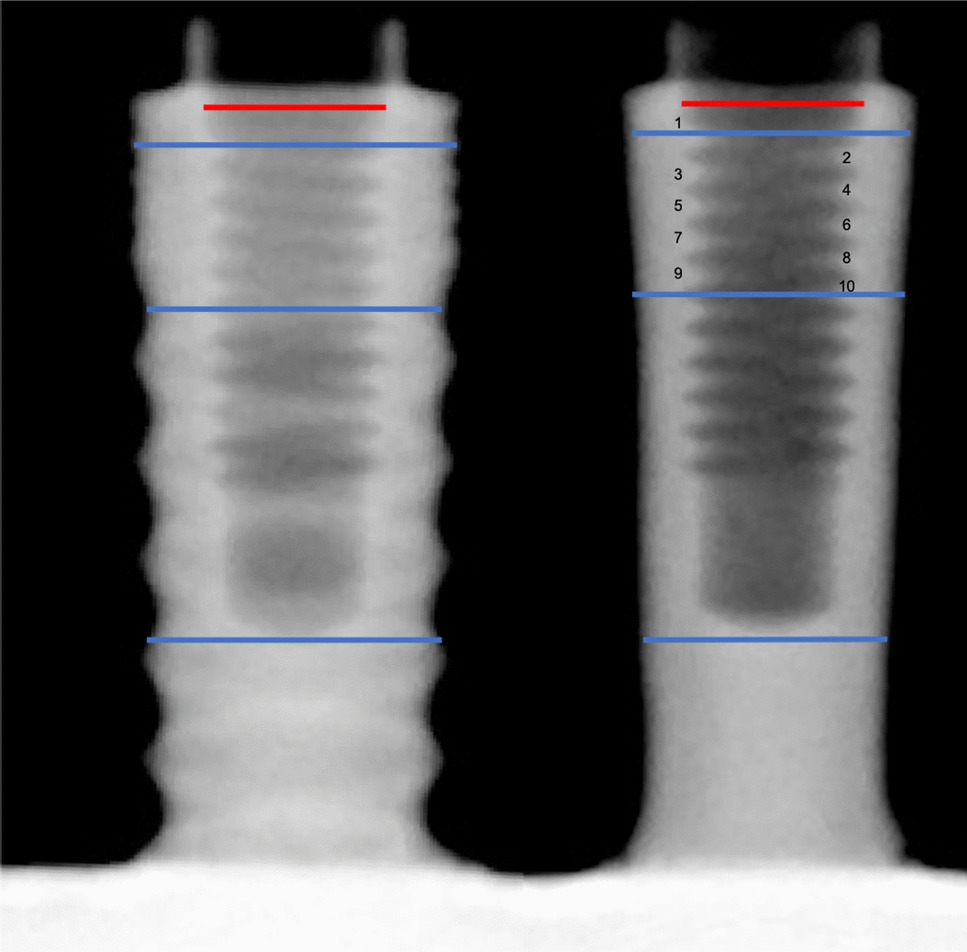
Radiographic measurements of the implant wall width. Left: control implant; right: IP implant. Blue lines: length at middle of the first (R1) and tenth (R2) threads and at the end of the prosthetic screw hole (R3), perpendicularly to the long axis of the implant; red line: 1.9 mm reference

The implant wall width was measured through plain x-ray examination of all the samples, in the initial position and rotated through 120° and 240°, using standardized mounts. All the measurements were made using ImageJ v.1.51 (National Institutes of Health, Bethesda, Maryland, USA), based on a fixed 1.9 mm reference provided by the manufacturer. A calibrated investigator (BLA) performed the examination with 400X amplification and searched for perforations of the implant walls. The measurements were made at the middle of the first (R1) and tenth (R2) threads and at the end of the prosthetic screw hole (R3), as shown in Fig. 2. To test intraexaminer agreement and consistency, the assessment of 6 randomly selected samples (54 measurements) was repeated after 2 weeks. The intraclass correlation coefficients were 0.96 (95% confidence interval (95%CI) 0.93–0.98; *P* < 0.001) and 0.96 (95% CI 0.92–0.98; *P* < 0.001), showing excellent reliability and consistency.

The mean value of the three measurements (rotation of 0°, 120° and 240°) was recorded for each location and implant. The measurements in the IP group were subtracted from those of their control analogues, thus obtaining the thinning of the implant for each variable.

### Fracture tests

**Fig. 3:**
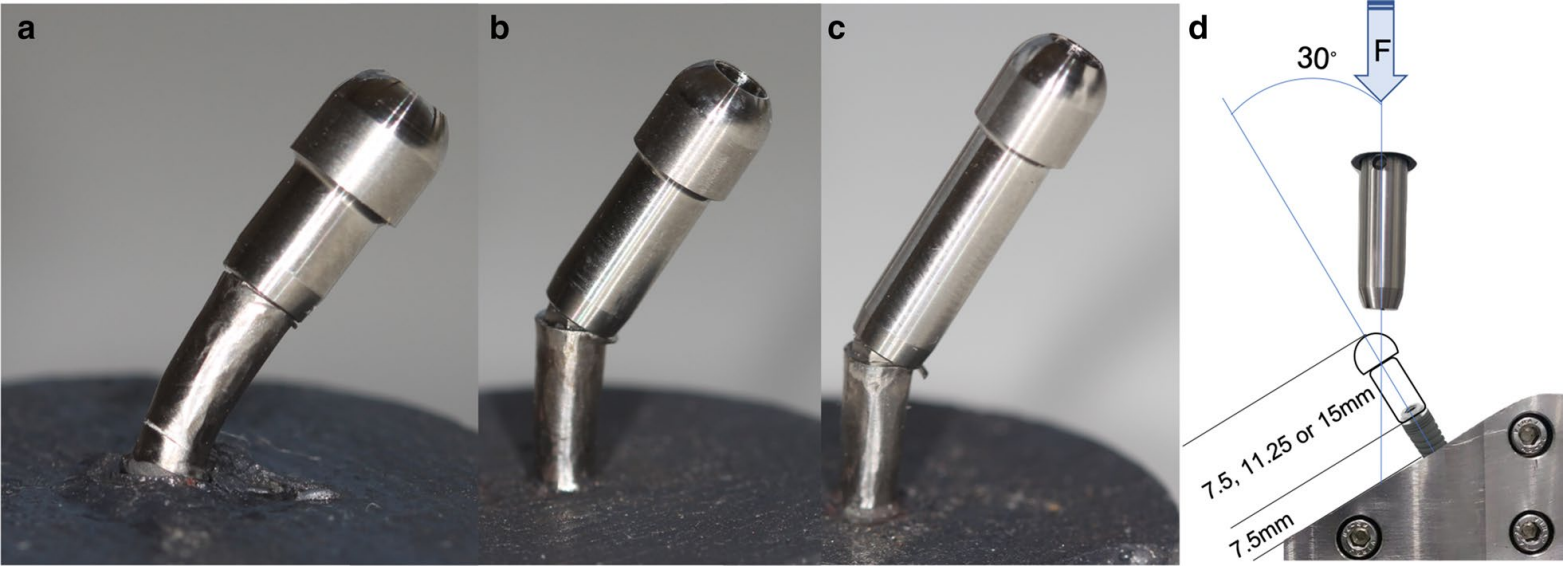
IP samples after fracture test: **a** CIR 2:1, **b** CIR 2.5:1, **c** CIR 3:1, **d** fracture test diagram

Metallic hemispherical load abutments (n = 48) were digitally designed, milled and screwed onto each implant according to subgroup (Fig. 3a–c), using prosthetic screws (Avinent® Implant System, Santpedor, Spain) at 32 N/cm, as recommended by the product manufacturer. Tests to measure the maximum compression force (F_max_), i.e. the maximum force reached before implant fracture, were performed at a constant speed of 1 mm/min with a universal servo-hydraulic mechanical testing machine (MTS Bionix 370 Load Frame, MTS®, Eden Prairie, USA), applying a compression load to the implants with a 661.19H-03 MTS Load Cell of 15 kN capacity. All the samples were held in the same device, a manufactured stainless-steel clamping jaw that allowed compression loads to be applied at a constant angle of 30º from the vertical axis (Fig. 3d), in accordance with ISO 14801:2016 (third edition), except for the supracrestal 50% of the total implant length. The tests were monitored by the MTS Flextest 40 Controller (MTS®, Eden Prairie, USA), which measured F_max_ and recorded real-time data.

Scanning electron microscopy (SEM) (Quanta 200®, FEI, Hilsboro, Oregon, United States) screening of the fractured implants was used to determine the fracture location.

### Statistical analysis

The sample size calculation was performed with Stata v.14 software (StataCorp®, College Station, USA). Considering F_max_ as the primary outcome measure, an analysis of variance with an α risk of 0.05 and a statistical power of 80% was performed. The mean fracture resistance values published by Gehrke [24] were used. Assuming a standard deviation of 500 N, the sample size was established as 8 implants per subgroup.

The implant characteristics were presented as absolute and relative frequencies for categorical outcomes. The normality of the scale variables (F_max_ and implant wall width) was explored using the Shapiro–Wilk test, P–P scatterplot graphs and box plots. Since F_max_ and the implant wall width variables had a normal distribution the mean and the standard deviation (SD) were used.

To analyze the effects of the procedure (IP or control) on F_max_, of the crown length (7.5, 11.25 or 15 mm), and of the interaction between these two variables, a two-way ANOVA was performed. The ANOVA assumptions were assessed using the Shapiro–Wilk test for normality and Levene’s test for homoscedasticity. Pairwise comparisons between subgroups, using Tukey’s correction for multiplicity of contrasts, were made for each procedure and CIR. An unpaired *t* test was used to identify differences in implant wall width between the control and IP groups at every reference point. In each area of interest, Pearson correlation coefficients were computed to quantify the correlation between implant wall width and F_max_. The associations between categorical variables were assessed with either Pearson’s χ^2^ test or Fisher’s exact test.

The statistical analysis was carried out with Stata14 software (StataCorp®, College Station, TX, USA). The level of significance was set at *P* < 0.05.

## Results

### Fracture tests

**Fig. 4:**
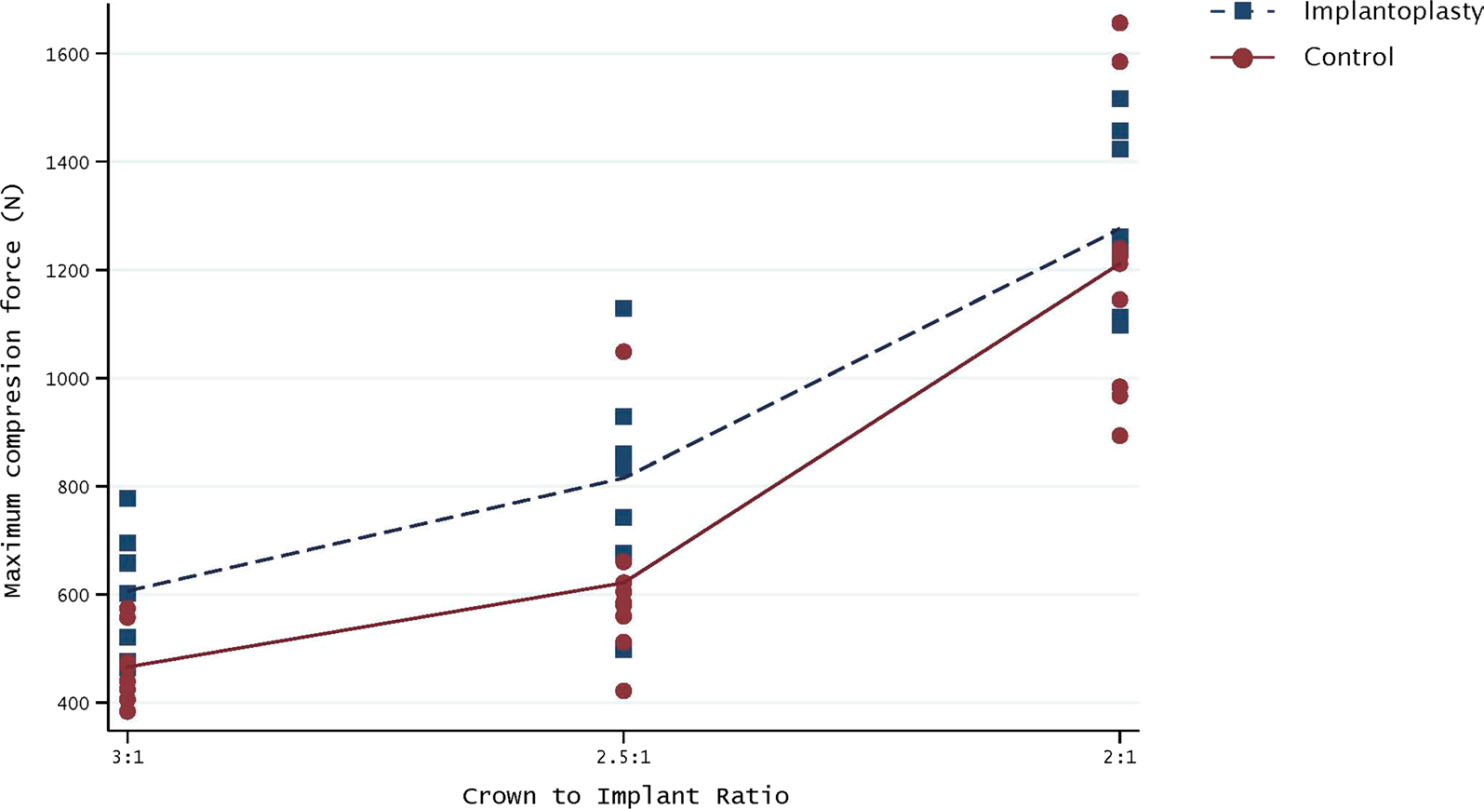
Mean fracture strength (N) of the three CIR ratios in the IP and control samples

No correlations between implant wall width measurements and F_max_ were observed at any of the reference points (Table 1). Significant reductions in F_max_ between the control and IP implants were only found in the 2.5:1 CIR subgroup (*P* = 0.037), although all the IP samples showed less resistance to fracture than their respective controls (Table 2, Fig. 4). In both IP and control groups, the implants with a 2:1 CIR showed a higher F_max_ (control = 1276.16 N ± 169.75; IP = 1211.70 N ± 281.64) than those with a 2.5:1 CIR (control = 815.22 N ± 185.58; IP = 621.68 N ± 186.28) and 3:1 CIR (control = 606.55 N ± 111.48; IP = 465.95 N ± 68.57). No significant differences were observed between the 2.5:1 and 3:1 subgroups (control *P* = 0.064; IP *P* = 0.206) (Table 3, Fig. 4).

**Table 1:** Implant wall width measurements (mm) of IP and control samples at each reference point (n = 48)

Reference point	Control	IP			
	Mean (SD)	Mean (SD)	MD (95%CI)	Independent samples *t* test *P* value	ANOVA *P* value
R1 (first thread)					
2:1	3.44 (0.02)	3.03 (0.04)	0.41 (0.37–0.44)	< 0.001^*^	0.103
2.5:1	3.44 (0.01)	3.03 (0.04)	0.41 (0.38–0.45)	< 0.001^*^	
3:1	3.45 (0.02)	3.08 (0.04)	0.37 (0.33–0.40)	< 0.001^*^	
R2 (tenth thread)					
2:1	3.32 (0.03)	2.86 (0.03)	0.46 (0.42–0.49)	< 0.001^*^	0.949
2.5:1	3.31 (0.02)	2.86 (0.04)	0.45 (0.41–0.49)	< 0.001^*^	
3:1	3.34 (0.03)	2.89 (0.06)	0.46 (0.41–0.50)	< 0.001^*^	
R3 (end of the prosthetic screw hole)					
2:1	3.07 (0.03)	2.62 (0.06)	0.45 (0.40–0.50)	< 0.001^*^	0.163
2.5:1	3.07 (0.05)	2.64 (0.04)	0.43 (0.38–0.47)	< 0.001^*^	
3:1	3.07 (0.02)	2.68 (0.04)	0.40 (0.36–0.43)	< 0.001^*^	

^*^Statistically significant difference

*MD* mean difference (Control—IP)

**Table 2:** Mean fracture strength (N) of the three CIR in the IP and control samples

CIR	Control	IP		
	Mean (SD)	Mean (SD)	MD (95%CI)	Adjusted *P* value
2:1	1276.16 (169.75)	1211.70 (281.64)	64.46 (− 117.17 to 246.09)	0.478
2.5:1	815.22 (185.58)	621.68 (186.28)	193.54 (11.91–375.17)	0.037^*^
3:1	606.55 (111.48)	465.95 (68.57)	140.60 (− 41.03 to 322.24)	0.126
Total	899.31 (323.58)	766.44 (379.19)	132.87 (− 71.95 to 337.69)	0.198

^*^Statistically significant difference

*MD* mean difference (Control—IP)

**Table 3:** Mean fracture strength (N) of the IP and control groups in the three clinical CIR subgroups

Group	CIR1	CIR2	MD (95% CI)	Adjusted *P* value
Control	2:1	2.5:1	460.94 (242.27–679.60)	< .001^*^
		3:1	669.60 (450.94–888.27)	< .001^*^
	2.5:1	3:1	208.67 (− 9.99 to 427.33)	.064
IP	2:1	2.5:1	590.02 (371.36–808.68)	< .001^*^
		3:1	745.75 (527.09–964.41)	< .001^*^
	2.5:1	3:1	155.73 (− 62.93 to 374.39)	.206
Total	2:1	2.5:1	525.48 (363.58–687.38)	< .001^*^
		3:1	707.68 (545.78–869.57)	< .001^*^
	2.5:1	3:1	182.20 (20.30–344.10)	< .001^*^

^*^Statistically significant difference

*MD* mean difference (CIR1—CIR2)

**Fig. 5:**
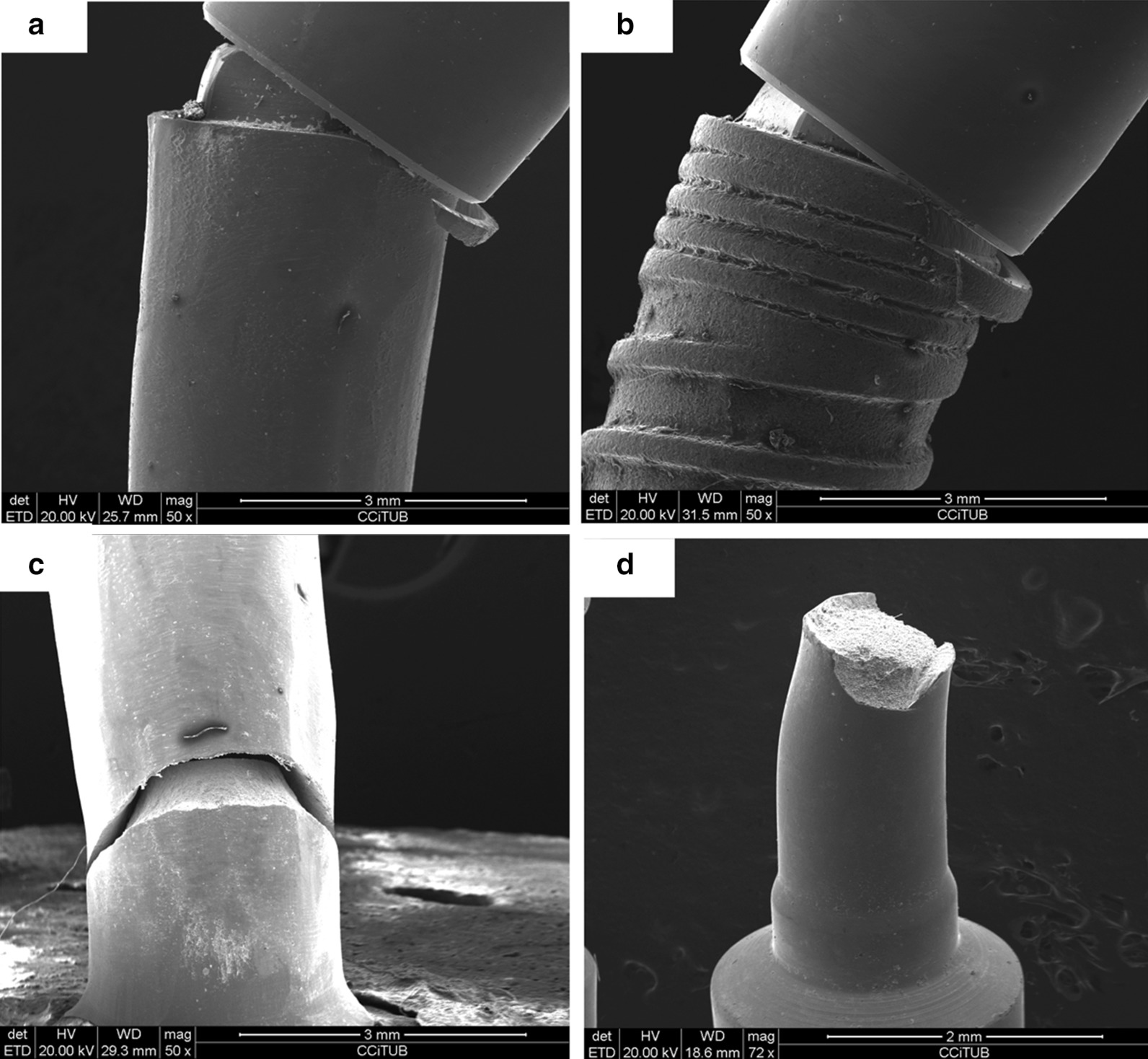
SEM screening: **a** IP sample platform fracture; **b** control sample platform fracture; **c** IP sample body fracture; **d** prosthetic screw fracture;

Most fractures (n = 43) were located in the platform area (Fig. 5a, b). The only 5 exceptions were found in implants with IP of the 2:1 CIR subgroup [4 fractures in the implant body (Fig. 5c) and 1 in the prosthetic screw (Fig. 5d)].

### Radiographic implant wall width measurements

The mean reduction in the implant wall width after IP was 0.41 (CIR 2:1), 0.41 (CIR 2.5:1) and 0.37 mm (CIR 3:1) at R1; 0.46 (CIR 2:1), 0.45 (CIR 2.5:1) and 0.46 mm (CIR 3:1) at R2 and 0.45 (CIR 2:1), 0.43 (CIR 2.5:1) and 0.4 mm (CIR 3:1), at R3 (Table 1). In all the subgroups, IP was associated with a statistically significant reduction in width at reference points 1–3 (*P* ≤ 0.05, independent samples *t* test) and a similar value was found at each reference point (*P* > 0.05 in all cases; one-way ANOVA) regardless of the crown length subgroup of the implant. No perforation of the inner threads of the implants were observed.

## Discussion

The main objectives of this in vitro study were to determine if narrow platform titanium implants with an external hexagonal connection subjected to IP were more prone to fracture in the presence of 50% bone loss, and to analyze if an increased CIR reduces the fracture resistance of implants with IP *vs.* control implants. The results of the present study show that IP only significantly reduced the F_max_ value in the 2.5:1 CIR subgroup. Besides, the mean total values of the 3 CIR subgroups showed no significant differences in F_max_ between the control and IP samples (Table 2). CIR seems to be a much more relevant variable than IP, since both the IP and control implants showed significant reductions in F_max_ in the 2.5:1 and 3:1 CIR subgroups when compared to the 2:1 subgroup (Table 3). Indeed, while IP reduced the mean fracture strength by 132.87 N, a higher CIR (2.5:1 or 3:1) led to a mean difference of 525.48 N or 707.68 N, respectively (Tables 2, 3).

Similar in vitro protocols have been described previously, although with different implants, bone insertion levels and loading abutments [15, 24–27]. Shemtov-Yona et al. used intact 13 mm-long implants with different widths and performed similar static tests, finding F_max_ values of 674 N ± 57 (3.3 mm implants), 952 N ± 103 (3.75 mm implants) and 1584 N ± 115 (5 mm implants), showing that implant wall width can affect resistance outcomes of intact implants [28]. On the other hand, Chan et al. using internal hexagonal implants, compared control and IP samples with different widths (3.75 and 4.7 mm) and showed that IP did not significantly affect the resistance to fracture of 3.75 diameter implants (321.7 N ± 21.4 vs. 325.0 N ± 20.7) [15]. The fact that our report presents higher F_max_ values (Table 2) might be considered surprising since the implant diameter was inferior (3.5 mm), the CIRs were unfavorable and the simulated bone level was of 50%. This discrepancy might be justified by the fact that our study employed external hexagonal implants which have shown higher F_max_ values in comparison with internal hexagonal implants in a recent published paper [26].

Significant differences in implant wall width due to the IP procedure were observed at all the reference points, but no perforations of the inner threads were found. The reduction in implant diameter at each of the 3 reference points ranged from 0.37 mm (95% CI 0.33–0.40 mm) to 0.46 mm (95% CI 0.41–0.50 mm) in the IP test samples. Other authors with similar IP protocols have reported lower reductions [25, 29]. These discrepancies might be explained by differences in the degree of polishing, but are more likely to be the result of different implant geometries, namely thread depth and model. Thus, further studies with different implants should be carried out, since their design and material are likely to affect the implant’s resistance to fracture. A similar extent of change was found at each reference point (*P* > 0.05 in all cases; one-way ANOVA), regardless of the crown length subgroup of the implant, showing the similarity of the IP across all these samples, which would indicate that the procedure should be easy to reproduce.

Previous reports have claimed that implant diameter affects stress fatigue behavior and that dental implants will attain a critical stress point at lower loadings when subjected to IP [15, 27, 28]. The present results corroborate this finding, as lower resistance to fracture was observed in the IP groups (Table 2). All the IP groups showed less F_max_ values than the control groups, although these differences were found to be significant in only one of the CIR subgroups (2.5:1). Hence, narrow platform implants seem to be structurally weakened by IP procedures, although the most relevant risk factor for mechanical complications in the presence of 50% of bone loss seems to be CIR, as the mean F_max_ values dropped to almost half between the 2:1 and 2.5:1 CIR subgroups (mean difference 590.02 N, 95% CI: 371.36 N to 808.68 N) and by 61.6% between 2:1 and 3:1 (mean difference 745.75 N, 95% CI: 527.09 N to 964.41 N) (Table 3). Bertl et al. [22] having obtained a statistically significant reduction of fracture resistance on IP implants, reported that the forces required to fracture or deform a narrow diameter implant with IP remained high and therefore, this reduction has a limited clinical relevance in the majority of cases.

In both the IP and control groups, F_max_ decreased with increasing CIR, although the only significant differences were between CIR 2:1 and the other two subgroups (Table 3). No significant differences between CIR 2.5:1 and 3:1 were observed despite the latter’s resistance to fracture being lower in both the IP and control implants (Control: 815.22 N vs. 606.55 N; IP: 621.68 N vs. 465.95 N). This outcome might be related with the limited sample size and with the observed standard deviations. However, it is important to stress that the lowest resistance value was found in the 3:1 CIR subgroup with IP (465.95 N ± 68.57).

In the present study, the area mostly affected by fracture was the platform, which would suggest that the platform is more fragile than the body in narrow fixtures. While all the control implants broke at the platform, in the IP group with a 2:1 CIR some fractures occurred in the body (n = 4) and prosthetic screw (n = 1), suggesting that IP reduces the mechanical resistance of the implant body. However, when higher CIRs were tested the stress seemed to be directed towards the platform and the prosthetic connection, and therefore all the fractures occurred in this area. Other studies using regular platform implants have found that implants subjected to IP usually break at the implant body, and although IP does not seem to decrease the maximum compression force of regular diameter external connection implants significantly, it clearly weakens the implant body [25]. Upon testing different CIRs with 3.5 mm intact external hexagon implants, fracture screw and implant platform deformation have been reported along with reduced resistance to fracture with increasing CIR. Gehrke performed an in vitro study with 60 implants with 3 different connections and also concluded that increasing the crown height significantly reduces the resistance to loading [24]. According to this paper, the abutment connection type also seems to be a relevant variable in the fracture resistance of dental implants, since morse taper implants seem to be less prone to fracture than external and internal hexagonal connections. However, IP may alter these results. Indeed, a recently published paper compared the fracture resistance after IP of three connection designs and concluded that external hexagonal connection implants have a higher resistance to fracture [26]. Another variable that should be taken into consideration is the degree of bone loss. This factor might be relevant since it affects the clinical crown height [30].

The present study presents some limitations related to its in vitro design. Firstly, the IP procedures were performed by hand to simulate real-life conditions, instead of using a milling machine. Although this might compromise the standardization of the implant reduction slightly, it increased the external validity of the outcomes. Secondly, long implants (15 mm) were selected in order to assure adequate retention in the resin during the fracture tests. The length and 50% exposure of the implant provide information especially for extreme bone loss cases. In addition, 3.5 mm wide implants were selected because previous reports have shown that narrower implants must be addressed carefully for IP [15]. Nevertheless, narrow implants are widely used and bone loss from PI can affect any implant. Consequently, these factors were considered valuable for understanding the threshold of fracture resistance. Although a 15 mm long implant with a 15 mm long restoration is not common, considering a bone level type implant it represents a standard 1:1 CIR. Also, when PI has caused the loss of 5 mm of bone, the 1:1 clinical CIR of a 10 mm long implant with a 10 mm long restoration becomes a clinical CIR of 3:1, similar to that of the 15 mm abutment subgroup in this study. In addition, the static compressive loads at a 30° angle used for fracture testing do not replicate the daily complex oral function of patients [31]. However, the methodology employed complied with ISO guideline 14801:2016 (third edition), except for the vertical exposure of the implant, allowing comparison with previous studies. Nevertheless, future research should include dynamic fatigue tests to determine the clinical relevance of the fracture resistance encountered. According to Gibbs et al., the maximum human clenching force covers a wide range, from 98 to 1243 N, and is affected by several factors including age, gender and tooth support [32]. The top of this range would fracture all the samples except for the controls with a 2:1 CIR [1276.16 N (σ = 169.75)].

Bite force seems to decrease from molar to premolar and to incisor. Maximum bite forces measured in male subjects are higher than those of female subjects according to Umesh et al. [33]. The same authors found maximum bite forces of 744 N in molars, 371 N in premolars and 320 N in incisors.

Considering the above outcomes and comparing them with the present data, IP procedures with a CIR of 2:1 (mean fracture strength 1211.70 N ± 281.64) would present a low fracture risk regardless of implant position, and fracture risk would be of concern after IP in molar regions with a CIR of 2.5:1 (mean fracture strength 621.68 N ± 86.28) or 3:1 (mean fracture strength 465.95 N ± 68.57).

In such cases, it would be advisable for clinicians to perform a risk–benefit analysis, since implant fractures are more likely to occur. Therefore, as the Young modulus of different titanium alloys and ceramic implants varies, further research is needed to determine the resistance to fracture of new materials used for dental implants.

## Conclusions

IP significantly reduces the fracture resistance of implants with a 2.5:1 CIR. The results also suggest that the CIR seems to be a more relevant variable when considering the resistance to fracture of implants, since significant reductions were observed when unfavorable CIR subgroups (2.5:1 and 3:1 CIR) were compared with the 2:1 CIR samples.

## Data Availability

The datasets used and analyzed during the current study are available from the corresponding author on reasonable request.

## Abbreviations

CIR: Crown-to-implant ratio
F_max_: Maximum compression force
IP: Implantoplasty
ISO: International Organization for Standardization
PI: Peri-implantitis
R1: Length at middle of the first thread
R2: Length at middle of the tenth thread
R3: Length at the end of prosthetic screw hole
SD: Standard deviation
SEM: Scanning electron microscopy
